# A Spatio-Temporal-Dependent Requirement of Sonic Hedgehog in the Early Development of Sclerotome-Derived Vertebrae and Ribs

**DOI:** 10.3390/ijms25115602

**Published:** 2024-05-21

**Authors:** Nitza Kahane, Yael Dahan-Barda, Chaya Kalcheim

**Affiliations:** Department of Medical Neurobiology, Institute of Medical Research Israel-Canada (IMRIC) and the Edmond and Lily Safra Center for Brain Sciences (ELSC), Hebrew University of Jerusalem-Hadassah Medical School, P.O. Box 12272, Jerusalem 9112102, Israel; nitzakahane@gmail.com (N.K.); yael.dahan3@mail.huji.ac.il (Y.D.-B.)

**Keywords:** BMP, dermomyotome, lateral plate mesoderm, myotome, neural tube, noggin, notochord, Pax7, somite, Sox9, sternum

## Abstract

Derived from axial structures, Sonic Hedgehog (Shh) is secreted into the paraxial mesoderm, where it plays crucial roles in sclerotome induction and myotome differentiation. Through conditional loss-of-function in quail embryos, we investigate the timing and impact of Shh activity during early formation of sclerotome-derived vertebrae and ribs, and of lateral mesoderm-derived sternum. To this end, Hedgehog interacting protein (Hhip) was electroporated at various times between days 2 and 5. While the vertebral body and rib primordium showed consistent size reduction, rib expansion into the somatopleura remained unaffected, and the sternal bud developed normally. Additionally, we compared these effects with those of locally inhibiting BMP activity. Transfection of Noggin in the lateral mesoderm hindered sternal bud formation. Unlike Hhip, BMP inhibition via Noggin or Smad6 induced myogenic differentiation of the lateral dermomyotome lip, while impeding the growth of the myotome/rib complex into the somatic mesoderm, thus affirming the role of the lateral dermomyotome epithelium in rib guidance. Overall, these findings underscore the continuous requirement for opposing gradients of Shh and BMP activity in the morphogenesis of proximal and distal flank skeletal structures, respectively. Future research should address the implications of these early interactions to the later morphogenesis and function of the musculo-skeletal system and of possible associated malformations.

## 1. Introduction

Ensuring the proper patterning of an embryo requires the coordinated operation of multiple processes throughout various developmental stages. A compelling illustration of this principle is provided by the development of the rib cage and its associated structures. Here, the interactions among developing muscles, connective tissue, ribs, and tendons orchestrate the creation of a scaffold crucial for the normal morphogenesis and function of the body wall [1,2,3]. The rib cage plays a vital role in providing postural support, safeguarding internal organs, and facilitating adequate ventilation. However, the mechanisms governing the formation and integration of these structures remain largely elusive.

Whereas vertebrae, ribs, and tendons derive from distinct subdomains of the somite-derived sclerotome [4,5], epaxial and hypaxial skeletal muscles derive from the medial and lateral dermomyotome (DM), respectively [6,7,8], intercalating into an early scaffold of pioneer myocytes stemming from the medial epithelial somite [9,10]. Hypaxial intercostal muscles accompanied by ribs expand into the somatic layer of the lateral plate mesoderm (LPM) where the distal rib connects with the forming sternum, a LPM derivative [11], thus establishing the thoracic cage.

Sonic Hedgehog (Shh), secreted from notochord and floor plate [12,13,14,15], is essential for the patterning and differentiation of neural [16,17] and mesodermal progenitors [18,19,20,21]. Using a mouse transgenic line, a ventro-dorsal activity gradient of Shh/Gli signaling was directly visualized, spreading from the notochord through the sclerotome [18]. In addition, in chick embryos, specific inhibition of Shh activity in sclerotome impaired DM cell proliferation and epitheliality and terminal differentiation of muscle progenitors that entered the myotomal domain [18]. Hence, the sclerotome serves as a pathway through which Shh is distributed to reach the DM and also acts to attain the neural tube through its basal domain to stimulate motoneuron development [18,22].

The ventral somite that generates the sclerotome also responds to Shh. Inhibition of Shh from the earliest stages of development, either through the loss of its signal-activating receptor, Smoothened, or by the absence of Gli transcription factors, results in compromised sclerotome specification, illustrated by lack of activation of crucial markers such as Pax1 and Pax9, etc., and leads to defects in axial skeleton formation [12,23,24,25]. Likewise, mouse mutants lacking Pax1 exhibit malformed proximal skeletal structures [26,27], and Pax1:Pax9 double mutants completely fail to form a sclerotome and consequently do not develop an axial skeleton [25]. Although midline-derived Shh is necessary both for myotome and sclerotome formation, direct myotome-sclerotome interactions, and subsequent intercostal muscle-rib communication, were also documented as being important for proper rib growth and patterning [28,29,30,31].

As anticipated for a morphogen displaying spatial and temporal dynamics, Shh may exert distinct effects on target cells, depending on the timing of its activity [32,33,34,35]. To explore potential differential effects, we decreased Shh levels by electroporating its negative modulator, Hedgehog-interacting protein (Hhip) [36,37], utilizing tetracycline-dependent conditional plasmids. Shh activity was disrupted in the ventral somite either continuously from day 2 to day 5 of development (E2–5), solely from E2 to E3, or from E3–5. Time-dependent effects on sclerotomal survival and proliferation, as well as later defects in proximal skeletal morphogenesis, were documented. These indicate that Shh is constantly necessary for various aspects of skeletal development throughout the period spanning from days 2 to 5 of quail development.

Additionally, given that Shh and BMP exhibit graded yet antagonistic activities in dorso-ventral patterning of the neural tube and medio-lateral patterning of the paraxial mesoderm [38,39,40,41,42,43], we compared these effects with those of locally inhibiting BMP activity. Initially, we observed that BMP activity, indicated by P-Smad 1,5,8 immunoreactivity, exhibits a decreasing latero-medial gradient in the LPM where it is synthesized. Notably, high P-Smad expression peaks in the ventrolateral lip (VLL) of the DM. Inhibiting BMP in the latter epithelium through Smad6 prompted cell-autonomous myogenic differentiation. Additionally, transfection of Noggin into the LPM hindered sternal bud formation and completely prevented the growth of the myotome/rib complex into the hypaxial body domain. These results confirm and further extend the notion that the lateral DM epithelium acts as a guidance cue for rib expansion into the body wall through a BMP-mediated mechanism.

## 2. Results

### 2.1. Decreasing Levels of Shh Activity in Sclerotome by Hhip1 Enhances Cell Death without Affecting Cell Specification

**Figure 1:**
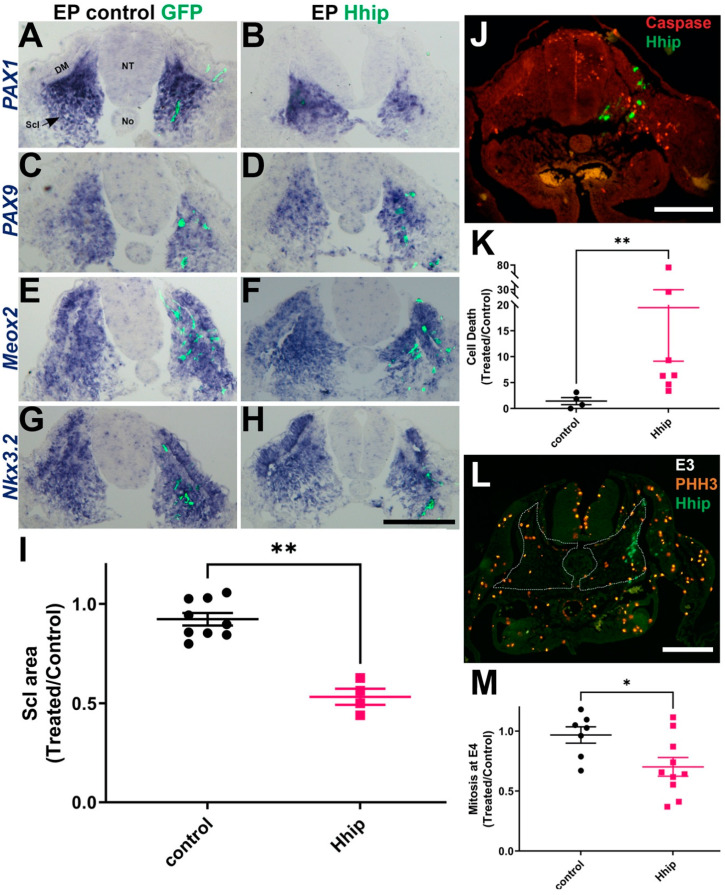
Reducing Shh activity in sclerotome reduces its size while enhancing cell death but does not affect cell specification (**A**–**H**) In situ hybridization for *PAX1*, *PAX9*, *Meox2*, *Nkx3.2* in embryos 14 h following electroporation to the ventral epithelial somite with control GFP (**A**,**C**,**E**,**G**) or Hhip (**B**,**D**,**F**,**H**). (**I**) Quantification of sclerotome (Scl) area. *n* = 9 and 4 embryos for control and Hhip, respectively. (**J**) Caspase immunostaining 14 h after electroporation at E2, showing the presence of caspase+ nuclei (red) in the Hhip-electroporated side (green). (**K**) Quantification of cell death, *n* = 4 and 7 embryos for control and Hhip, respectively. (**L**) Cell mitosis at E3 following electroporation at E2, visualized by PHH3 staining (red); no differences are apparent between treated and control sides. (**M**) Quantification of cell proliferation at E4, 2 days post-electroporation. *n* = 7 and 10 embryos for control and Hhip, respectively. Abbreviations, EP, electroporation, DM, dermomyotome; No, notochord; NT, neural tube. * *p* < 0.05; ** *p* < 0.01. Bar = 100 μM.

We examined the effects of Shh deprivation on early sclerotome development, by misexpression of the high affinity and selective Shh antagonist H hip1 [18]. Ventral halves of flank-level epithelial somites were focally electroporated in embryos aged 23–25 somite pairs. A day later, the sclerotomal markers *PAX1*, *PAX9*, as well as *Meox2* and *Nkx3*.*2* that additionally identify the DM, were normally expressed in treated compared to control sides or to control GFP-treated embryos (Figure 1A–H) [44,45,46,47,48]. However, the treated sclerotomes exhibited a notable reduction in size (Figure 1A–I), along with a decrease in the adjacent DMs (Figure 1F,H), consistent with previous findings [18,49].

Notably, staining for apoptotic cells via caspase revealed increased cell death within the Hhip-treated segments (Figure 1J,K). Interestingly, no immediate impact on cell mitosis was observed, although a reduction in mitotic figures became evident two days post-transfection (Figure 1L,M). Consequently, the reduction of Shh during the epithelial somite stage predominantly influences early sclerotomal cell survival, resulting in diminished sclerotome size, while leaving cell specification unaffected. Thus, this experimental approach enables an analysis of sclerotome derivative morphogenesis independently of initial patterning.

### 2.2. Reducing Shh Activity within the Sclerotome at Different Stages Yields Varied Effects on Skeletal Morphogenesis

**Figure 2:**
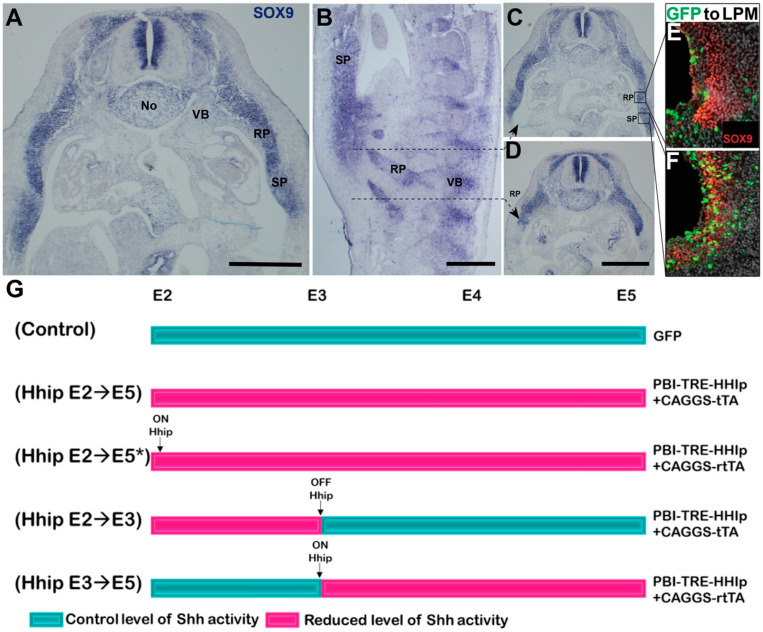
Skeletal components apparent in the flank region of quail embryos at E5. (**A**–**D**) Transverse (**A**,**C**,**D**) and sagittal (**B**) sections in situ hybridized for *SOX9*, showing, at E5, the presence of vertebral bodies (VB) surrounding the notochord (No), rib primordia (RB) entering the LPM, and sternal primordia (SP) ventrally located. (**E**,**F**) Electroporation at E2 of control GFP (green) to the LPM reveals in (**E**); no contribution to the Sox9-immunoreactive RP (red), yet significant colonization of the SP with cells co-expressing GFP and Sox9 (**F**). (**G**) Schematic representation of the experimental protocol implemented for conditional Hhip misexpression. * denotes the second method for Hhip misexpression between E2–5. See Results and Methods for further details. Bar = 300 μM.

By E5 in control embryos, the sclerotome has undergone differentiation into a vertebral body (VB) surrounding the notochord, and a rib primordium (RP) extending into the somatic mesoderm layer of the LPM. At this stage, the rib primordium, identified by *SOX9* expression, represents the initial stages of rib formation, with no distinct separation into proximal and distal (sternal) parts yet discernible (Figure 2A–D). Furthermore, a ventral bud was identified as the sternal primordium (SP). Unlike ribs, it lacked segmental patterning, covered only a portion of the rib extent, and notably, contained cells co-expressing Sox9 and GFP upon electroporation of the coelomic membrane, indicating its origin from the LPM (Figure 2C–F).

We monitored the effects of reduced Shh activity by overexpressing a control GFP plasmid, or tetracycline-inducible plasmids encoding Hhip1. To inhibit Shh signaling between embryonic days E2 and E5, we utilized either PBI-TRE-Hhip+CAGGS-tTA (Tet-on, an active plasmid unless doxycycline is added) or PBI-TRE-Hhip+rtTA2s-M2 (*Tet-off, an inactive plasmid unless doxycycline is added), followed by immediate soaking with doxycycline (Figure 2G) [18,50]. Since these plasmids elicited similar effects across all parameters tested, results were combined (Supplementary Figure S1). To reduce Shh signaling between E2 and E3, or between E3 and E5, a similar approach was implemented, as shown in Figure 2G. Electroporation was performed at E2, and embryos were sacrificed at E5 in all instances.

**Figure 3:**
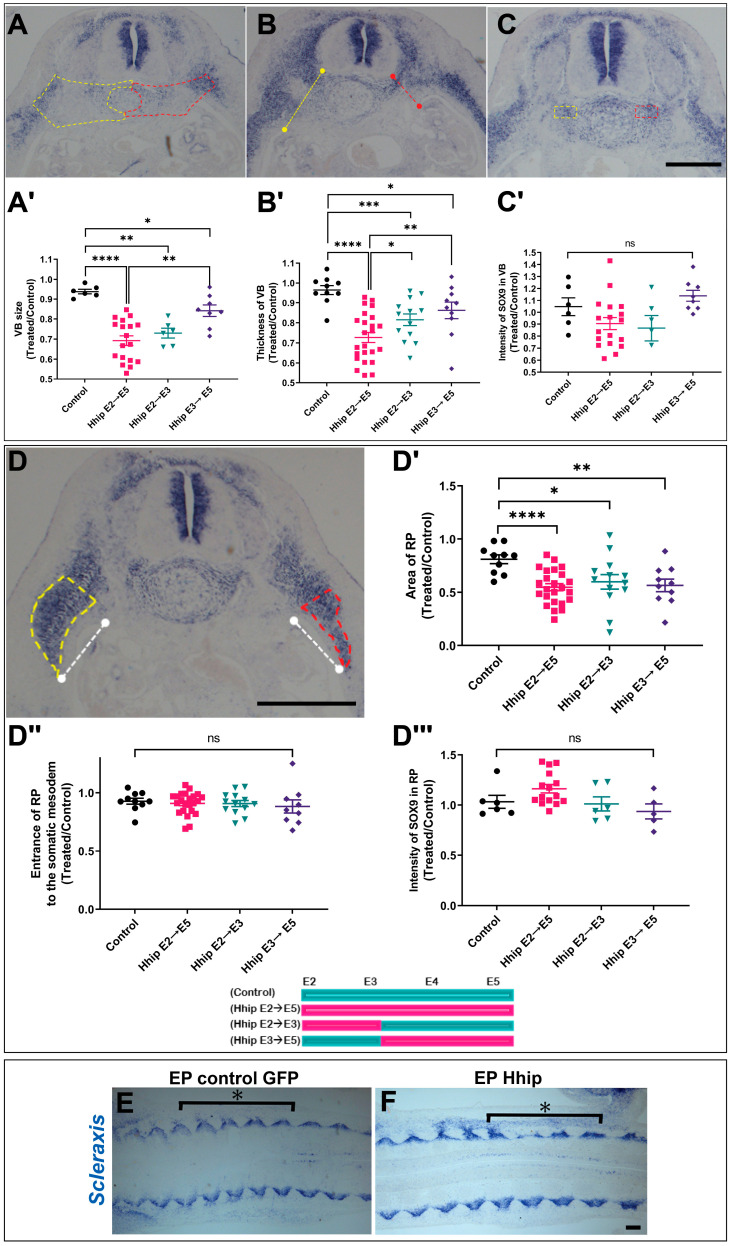
Effects of conditional Shh inactivation on development of various skeletal components (**A**–**D**) E5 transverse sections in situ hybridized for *SOX9*, depicting measurements of VB size (**A**), VB thickness (**B**), intensity of *SOX9* in VB (**C**), and RP size; extent of entrance into the LPM; and intensity of *SOX9* in RP (**D**); performed in treated (red) compared to control sides (yellow). (**A′**,**B′**,**C′**,**D′**,**D″**,**D‴**) Quantification of the above parameters as depicted in each graph. See Methods for further details. *n* = 6–24 embryos per treatment were analyzed as shown in the scatter plots. * *p* < 0.004, ** *p* < 0.002, *** *p* < 0.0003, **** *p* < 0.0001, ns, not significant. (**E**,**F**) Frontal sections in situ hybridized for *Scleraxis* with asterisks depicting electroporated region. Bar = 300 μM.

Initially, we assessed the impact of Shh deprivation on VB size (Figure 3A,A′). While all treatments resulted in a significant reduction, depriving Shh between E2 and E3 produced a similar effect to depleting it throughout the entire period. To note is that the later treatment (Hhip from E3 to E5) had a smaller yet still meaningful effect. Additionally, all treatment regimes reduced the dorso-ventral thickness of the VB when compared to the control side, with a smaller difference between the shorter deprivation periods (Figure 3B,B′ and see legend of Figure 3 for details). In contrast, neither treatment revealed a noteworthy difference in the intensity of *SOX9* expression within the VB (Figure 3C,C′). Together, these findings suggest that Shh is continuously necessary for regulating VB size but not for the progression of skeletal differentiation. However, VB size appears particularly sensitive to the loss of Shh signaling during the earliest stage that encompasses the period between epithelial somite and initial sclerotome formation.

Subsequently, we examined the development of the RP. Activating Hhip throughout the entire period from E2 to E5 resulted in a similar reduction in RP size, as observed with limited activations of Hhip over specific timeframes. Despite its diminished size, the RP invaded the somatic mesoderm to a comparable extent, and the intensity of SOX9 expression remained unchanged (Figure 3D–D‴). Frontal sections at the VB level in situ hybridized for *Scleraxis*, a marker of the syndetome, confirmed the above observations (Figure 3E,F). These findings highlight the continuous requirement for Shh signaling to regulate the size of the developing RP while not influencing its differentiation. Remarkably, the ability of the RP to invade the hypaxial domain of the body wall seems independent of rib size.

We observed increased cell death when Shh signaling was disrupted between E2 and E3 of development (Figure 1). Given that later treatments (E3 to E5) also affected VB and RP growth (Figure 3), we investigated cell proliferation and death upon Hhip activation at E3 for a duration of 14 h. Consistent with the continuous requirement for Shh signaling, the sclerotome area and the ratio of mitotic cells were decreased on the treated compared to the control side. However, at this stage, virtually no caspase-positive cells were observed on either treated or control sides (Supplementary Figure S2). Thus, while the initial effects of Shh deprivation may primarily stem from increased cell death, defects in growth observed upon later exposure to Hhip can be attributed to reduced cell proliferation.

**Figure 4:**
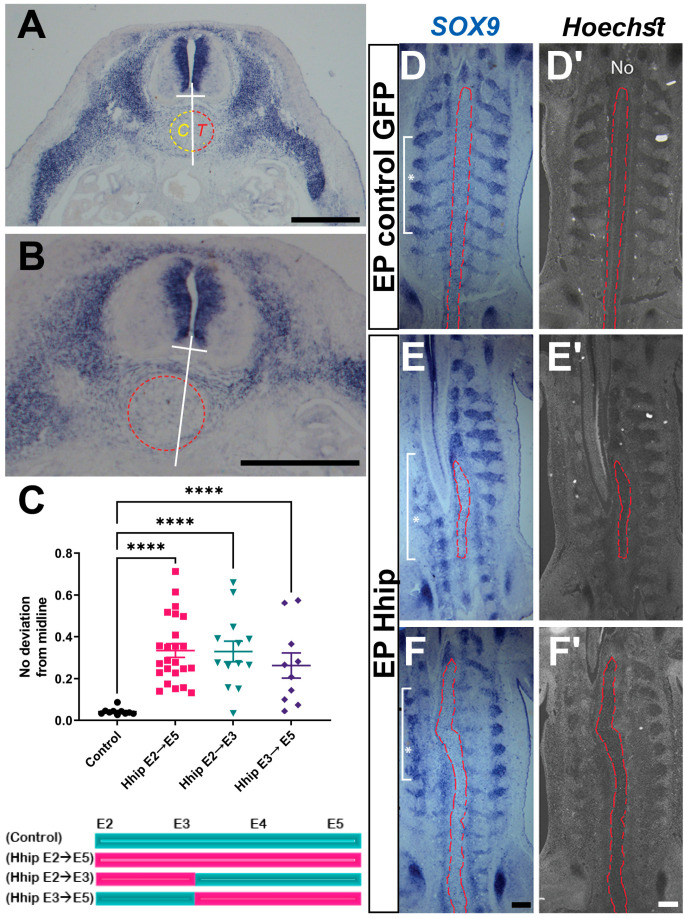
Notochord deviation from the midline in embryos with reduced Shh activity. (**A**,**B**) Transverse sections at E5 in situ hybridized for *SOX9* depicting the mode of measurement of notochord deviation comparing treated (area of notochord defined by red stippling) with control (area of notochord surrounded by yellow stippling) in control (**A**) and treated (**B**) embryos. See Methods for further details. (**C**) Quantification of notochord deviation showing significant effects of each experimental paradigm compared to control. *n* = 10, 24, 13, and 10 embryos analyzed for control, or Shh deprivation between E2–5, E2–3 or E3–5, respectively; **** *p* < 0.0001. (**D**–**F**) Frontal sections in situ hybridized for *SOX9* (**D**–**F**) and counterstained with nuclear Hoechst (**D′**–**F′**). A straight notochord is apparent in control (**D**,**D′**), whereas undulated notochords are seen in presence of Hhip (delimited by red dashed area). Electroporated region delimited by a white line and *. This sometimes resulted in frontal sections traversing both neural tube and notochord along the rostro-caudal axis (**E**,**E′**). Bar = 300 μM.

An additional prominent phenotype observed in embryos with deprived Shh activity was the deviation of the notochord from the ventral midline (Figure 4A,B). Even with unilateral electroporation, the notochord or part of it exhibited random deviations towards either side. This deviation was consistently significant across all treatments compared to controls (Figure 4C) and was also evident in frontal sections through the notochord level (Figure 4, compare E,F with D). Therefore, it is likely that Shh activity plays a crucial role in maintaining the rigid, rod-like structure characteristic of the notochord.

### 2.3. Differential Effects of Shh and BMP on Skeletal Development

It has been shown that Shh and BMP activities are necessary for the development of proximal and distal skeletal derivatives, respectively (reviewed in [11]). We revised this notion by performing focal electroporations of Hhip to abrogate Shh signaling or of Noggin/Smad6 to inhibit BMP activity.

**Figure 5:**
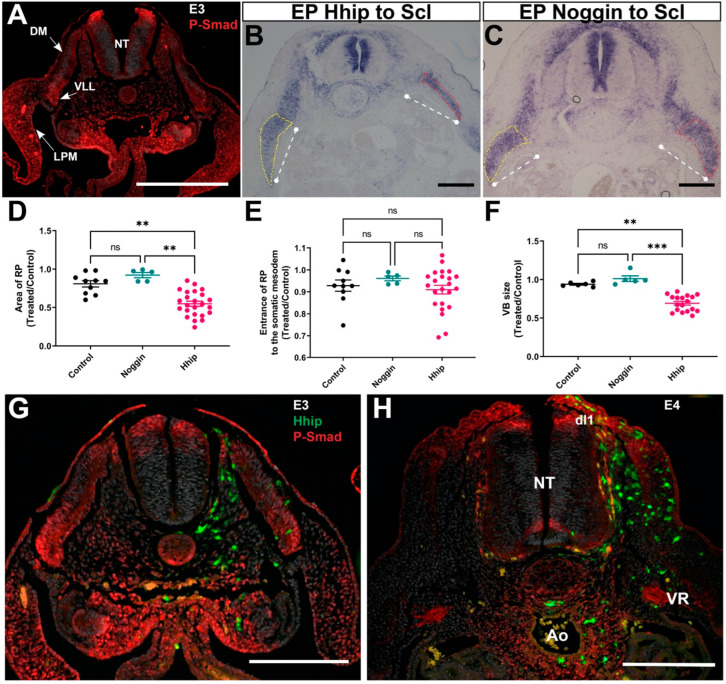
Unlike reduction of Shh activity, inhibition of BMP signaling in sclerotome does not affect skeletal development. (**A**) BMP activity at E3 illustrated by P-Smad immunostaining. Note a ventral to dorsal decreasing gradient in LPM (arrow), activity in the para-aortic region, in the dorsal neural tube (NT), and in the VLL of the DM epithelium (arrow). (**B**,**C**) Transverse sections at E5 stained for *SOX9* mRNA following electroporation of either Hhip (**B**) or Noggin (**C**) to the prospective sclerotome at E2. Treated sides are to the right and RP encircled by red or yellow stippling, respectively. The white straight lines delimit the extent of RP entrance into the LPM. (**D**–**F**) Quantification of RP area; extent of RP entrance into the LPM and VB size. Whereas Hhip significantly reduced RP and VB size, it had no effect on LPM ingression. Noggin was without effect on all parameters tested. *n* = 10, 5, 24 embryos examined for control, Noggin, and Hhip treatments. ** *p* < 0.01, *** *p* < 0.001, ns-not significant. (**G**,**H**) P-Smad immunostaining at E3 (**G**) or E4 (**H**) following unilateral Hhip transfection into sclerotome at E2. Note similar patterns of BMP activity at E3 between treated and control sides and compared with an intact embryo (**A**). Likewise, at E4, P-Smad was similarly expressed on both sides in dorsal interneurons (dIs), ventral neural tube (NT), ventral roots (VR), and para-aortic (Ao) area. Bar = 200 μM.

Whereas Shh is exclusively produced in notochord and floor plate and exerts its effects at a distance in all directions [38,51,52], BMP4 is synthesized in dorsal neural tube [53,54] and LPM [55]. Consistently, staining for P-Smad 1,5,8, a marker for BMP activity, was detected at E3 in the dorsal neural tube, in a decreasing ventral to dorsal gradient in the somatic layer of the LPM, and, surprisingly, also peaks in the VLL of the DM epithelium (Figure 5A and see also G).

Whereas transfection of Hhip into the ventral somite at E2 caused a significant reduction in VB and RP size at E5, Noggin electroporation to sclerotome had no effect on either structure considered. The magnitude of RP extension into the LPM was, however, unaffected by either treatment (Figure 5B–F and see also Figure 3). In addition, no change in the patterns of P-Smad activity was detected in the Hhip-transfected embryos at E3 or E4 (Figure 5G,H), suggesting that there is no compensatory interaction between both morphogens at the stages examined, differing from earlier stages [56].

**Figure 6:**
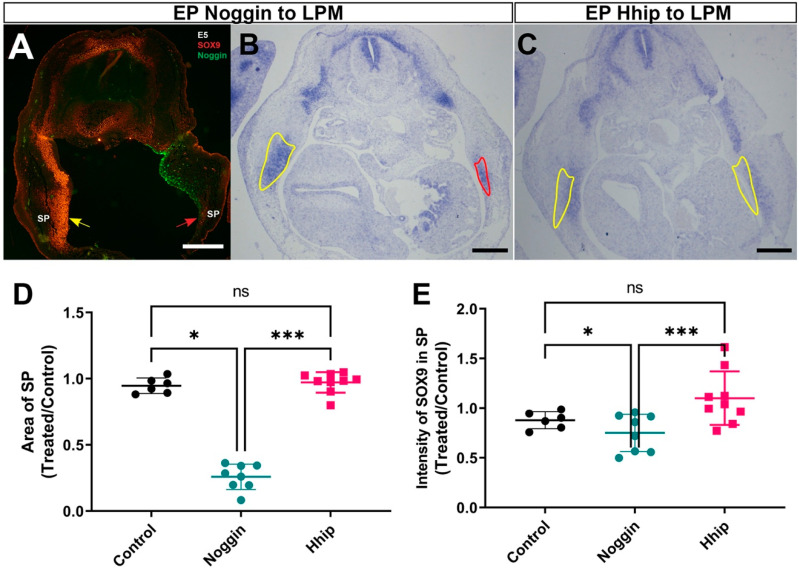
Inhibition of BMP activity in the LPM, unlike abrogation of Shh activity, reduced the size of the sternal primordium (**A**–**C**) Transverse sections at E5 of embryos unilaterally electroporated with Noggin (**A**,**B**) or Hhip (**C**) to the LPM. In (**A**,**B**), note dramatic reduction of the SP revealed by Sox9 immunostaining (**A**, red arrowhead vs. yellow arrow) or in situ hybridization [(**B**), area encircled by a red line compared to control SP delimited by yellow]. (**C**) Hhip has no effect on SP size. (**D**,**E**) Quantification of SP area and *SOX9* intensity showing reduced size only upon Noggin treatment. *n* = 6, 8, 9 embryos for control, Noggin or Hhip, respectively. * *p* < 0.05, *** *p* < 0.001, ns, not significant. Bar = 300 μM.

In contrast to electroporations targeted at the ventral somite, when either Hhip or Noggin were missexpressed in the coelomic epithelium that generates the LPM, the size of the SP was dramatically reduced in absence of BMP activity, whereas it developed normally in cases treated with Hhip. In contrast, the intensity of *SOX9* expression was not altered (Figure 6). Thus, the results of spatially restricted loss of factor activities confirm the accepted notion that Shh is required for development of proximal skeletal structures, whereas BMP is necessary for development of the distally forming sternum.

### 2.4. The VLL of the DM Is Responsible for Ingression of the RP into the Somatic Mesoderm

**Figure 7:**
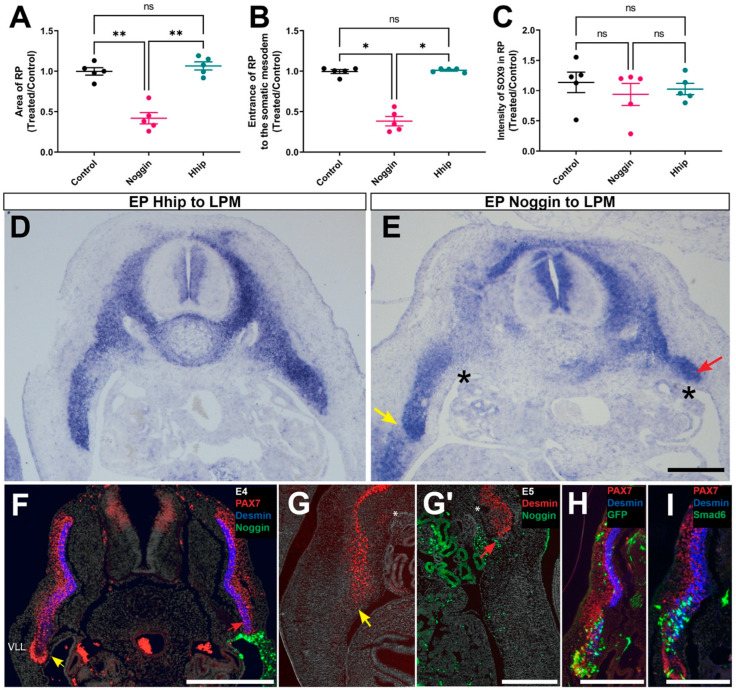
Reduced RP size and ingression into the LPM due to BMP deficiency are linked to hypaxial muscle growth failure through direct impact on the lateral DM epithelium. (**A**–**C**) Quantification of RP area (**A**), extent of entrance into the LPM (**B**), and intensity of SOX9 expression in E5 embryos electroporated at E2 with either Noggin or Hhip to the LPM. Only Noggin caused a significant reduction of RP size and LPM ingression. *n* = 5 embryos for each treatment). * *p* < 0.05, ** *p* < 0.0, ns, not significant. (**D**,**E**) Transverse sections showing, in (**D**), no effect of Hhip on the measured parameters, and in (**E**), a control RP that invaded the LPM in close association to the coelomic membrane (yellow arrow, left side), whereas upon Noggin transfection (right side), a smaller RP stopped close to the upper border of the coelomic cavity (*) and failed to progress ventralward (red arrow). (**F**) Transverse section at E4 illustrating the presence of a VLL (yellow arrow) in the control side but no VLL (red arrow) in the Noggin-treated side where desmin immunoreactive muscle (blue) reaches the distal tip. (**G**,**G′**) Transverse sections through an embryo at E5 illustrating the ingression of the control myotome into the LPM (**G**, yellow arrow) and the myotome in the treated side remaining in the epaxial-hypaxial boundary (**G′**, red arrow, * depict the upper coelomic border). (**H**,**I**) Electroporation of control GFP into the dorso-lateral somite at E2 displays, 2 days later, the presence of many GFP+ cells in the VLL, while others colonize the myotome. (**I**) Upon Smad6 transfection, virtually all labeled VLL progenitors exit the Pax7+ epithelium and differentiate into desmin+ muscle. (**H**,**I**), Bar = 200 μM; (**D**–**G′**), 300 μM.

**Figure 8:**
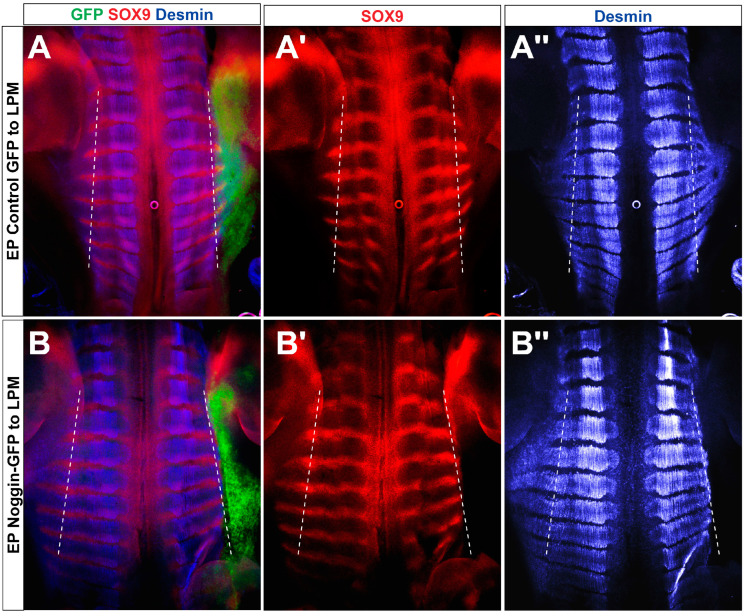
Rib primordia and hypaxial muscles fail to enter the LPM in absence of BMP signaling. (**A**,**B**) E5 embryos electroporated at E2 with either control GFP (**A**–**A″**) or Noggin-GFP (**B**–**B″**) to the LPM. Following fixation and immunostaining, embryos were eviscerated and prepared as an open book with their medial side pointing laterally. Images depict dorsal views of the wholemount preparations stained for GFP, Sox9, and desmin, illustrating, in controls, the entrance of both RP and hypaxial myotomes into the LPM (co-expression of GFP/desmin/Sox9 lateral to the dotted lines). In contrast, inhibition of BMP signaling in LPM prevented the ingression of both ribs and hypaxial muscles into the somatic mesoderm compared either to the intact contralateral side or to control GFP-treated embryos (note, in the treated side in (**B**–**B″**), no overlap between Sox9/desmin and GFP, e.g. no ribs or muscle tissue apparent lateral to the dotted line, see also Figure 7 for transverse sections). Dotted lines mark the epaxial/hypaxial boundary. *n* = 6 and 16 embryos for control and Noggin, respectively, that showed a similar phenotype. Rostral is to the top.

Consistent with Shh being an axial-derived factor, Hhip missexpression in LPM had no effect on the size of the RP or its ingression into the LPM (Figure 7A–D). In contrast, electroporation of Noggin into the coelomic epithelium, close to the site of BMP production and activity, markedly decreased the size of the RP without affecting the intensity of *SOX9* expression in the remaining tissue (Figure 7A–C,E). Most significantly, it prevented the RP from invading the hypaxial domain of the body, with the distal tip of the RP unable to progress beyond the dorsal aspect of the coelomic cavity (Figure 7E, arrow and asterisk, Figure 8). It is noteworthy that Noggin electroporation into the LPM also led to the loss of the Pax7+ VLL of the DM epithelium with expression of desmin reaching the ventral-most region (Figure 7F, red arrow), and neither the desmin+ myotome (Figure 7F and G′ compared to G, red arrows), nor the rib bud (Figure 7E), invaded the hypaxial LPM (see also Figure 8 for wholemount views).

Since the dermomyotomal VLL is a site of high BMP activity (Figure 5A,G) even if the factor is not locally synthesized, and that it disappears upon Noggin transfection to the LPM (Figure 6), we next examined whether the loss of the VLL in absence of BMP results from a direct effect on the epithelium. For this purpose, we electroporated the BMP inhibitor Smad6 directly into the dorso-lateral somite that develops into the corresponding part of the DM. Whereas control GFP-expressing cells both entered the lateral myotome as desmin-positive myocytes and also remained in the VLL epithelium as myogenic progenitors co-expressing Pax7 [7], virtually all Smad6-treated epithelial cells exited the VLL epithelium and cell-autonomously differentiated into lateral myocytes co-expressing GFP and desmin (Figure 7H,I). Collectively, BMP activity emanating from the hypaxial LPM also acts in the lateral epaxial domain to directly maintain the epithelial state of the VLL and prevent premature myogenesis. In the absence of BMP, the differentiating muscle fibers, being post-mitotic [9,57], fail to extend into the body wall, likely impeding the concurrent ingression of the accompanying RP.

## 3. Discussion

The effects of Shh on skeletal formation were mostly addressed in mutants of Shh or of downstream components of the pathway [12,26,27]. These studies demonstrated that Shh induces expression of early sclerotome markers such as *Pax1*, *Pax 9*, *Nkx3.2*, and *Mfh1* and affects later development of proximal skeletal derivatives (reviewed in [58]). By implementing targeted misexpression of the specific Shh inhibitor Hhip at various times and locations, we addressed the hypothesis that the effects of reduced Shh activity on skeletal morphogenesis are complex and can vary depending on the timing and specific context of Shh signaling disruption. We here show that reducing factor activity at the epithelial somite stage did not affect expression of early sclerotomal markers but significantly reduced sclerotomal size, a process accounted for by enhanced cell death. Moreover, decreasing Shh activity a day later in the sclerotome had no effect on cell death yet reduced cell proliferation. Thus, although enhanced cell death can mask underlying changes in proliferation, we here suggest that the effects on survival and cell proliferation by Shh are likely to be sequential and even transduced by different downstream mechanisms [59,60]. Our results refine the interpretation of a previous study performed in chick embryos claiming a survival role for Shh based on surgical ablation of the neural tube and notochord that strongly affected epaxial muscle, vertebrae, and rib formation due to somitic cell death [61].

**Figure 9:**
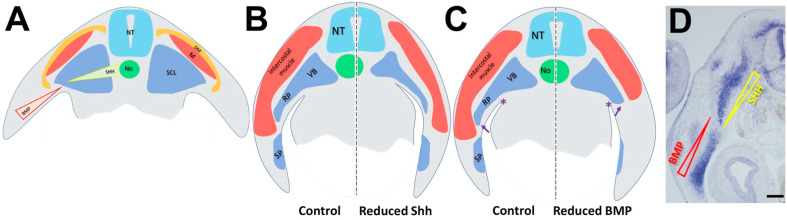
A schematic model for sclerotome and LPM-derived early skeletal development. (**A**) At early stages, counter-gradients of notochord-derived Shh and LPM-derived BMP pattern the medio-lateral identity of the paraxial mesoderm and account for early development of sclerotome and myotome. (**B**) Reduction of Shh signaling in sclerotome affects muscle differentiation and causes smaller vertebral bodies and rib primordia without affecting sternum development. (**C**) Compromised BMP activity in LPM prevents sternal development and hinders the progression of the myotome/rib complex into the LPM (arrows). * denotes the upper border of the coelomic cavity. (**D**) Medial Shh and latero-ventral BMP operate also at later stages to account for selected aspects of skeletal development. Bar = 200 μM.

In addition, our results confirm and extend previous genetic data on the requirement for Shh in development of proximal skeletal components such as the VB and RP but not of the LPM-derived SP [12,62] (Figure 9). We document that either early (E2 to E3) or somewhat later (E3 to E5) factor deprivation similarly impacts vertebral or rib size, suggesting a constant requirement for Shh between the epithelial somite stage and the time of formation of skeletal primordia. In contrast, the extent of ingression of the somite-derived RP was in all tested paradigms independent of its size, suggesting that invasion of the hypaxial somatic mesenchyme depends instead on the LPM providing a permissive substrate [63].

In addition to disturbed paraxial mesoderm derivatives, reduction of sclerotomal Shh also caused a misalignment and deviation of the notochord from the midline, suggesting it is necessary for structural integrity and positioning of the latter. Not surprisingly, being produced by the notochord, Shh was shown to be necessary for the formation of the sheath encircling the notochord itself. Removal of Shh signaling resulted in the formation of small and dispersed nuclei pulposi, remnants of the primitive notochord that localize to the middle part of the intervertebral disks [64]. Our findings also resemble a phenotype observed in conditional integrin β1 mutants, which exhibited disrupted and displaced notochords [65]. Given that Shh contributes to the establishment of a laminin-containing basement membrane in myotomes [reviewed in [66]] it is tempting to propose that by influencing the production of laminin, which acts as an integrin ligand, Shh also orchestrates the formation of the notochord sheath.

Lateral patterning of the paraxial mesoderm is induced by BMP signaling from the LPM [42,67] and treatment with BMP is able to generate LPM from segmental plate tissue [68]. Supporting this, both BMP4 mRNA [55] and BMP activity (this study) exhibit a decreasing ventro-dorsal gradient within the somatic layer of the LPM. Intriguingly, we observe that BMP activity reaches its peak in the VLL of the DM, despite the neighboring area showing low activity, and even though this region does not produce BMP family members (such as BMP4, 7, or 2) [55]. The precise mechanism underlying the heightened responsiveness of the VLL to BMP remains unclear. It is possible that the VLL responds to BMPs produced in the overlying ectoderm, albeit inhibition of BMP in the LPM with no ectodermal involvement profoundly affected this epithelium. In this context, it is worth noting a similar pattern of Shh/Gli activity in mice, where a diminishing medio-lateral gradient was identified in the sclerotome, with a peak of activity observed in the myotome, distant from the source of factor synthesis [18]. In this scenario, positive modulators of Shh activity were produced in the myotome, suggesting that low levels of the factor can translate into high activity levels in the presence of appropriate pathway regulators. Whether a similar situation applies for BMP signaling vis-a-vis the lateral DM remains to be examined. Along this line, Shh signaling impacts expression of FGF8 in the myotome, including its hypaxial domain, and misexpression of FGF8 causes rib abnormalities [11,29]. Thus, myotomal FGF8 might integrate signals emanating from Shh and BMP to modulate aspects of vertebral formation.

In this context, we demonstrate that the growth of the hypaxial myotome relies on the epithelial integrity of the VLL of the DM. Inhibition of BMP in the LPM via Noggin resulted in the loss of the VLL epithelium and prevented the expansion of the myotome into the body wall. Our results are consistent with those of Sudo et al., who grafted Noggin-expressing COS cells dorsal to the intermediate mesoderm and similarly observed that the sternal part of the ribs, as well as the hypaxial myotome, failed to penetrate the LPM [55]. Additionally, we show that the VLL epithelium directly responds to BMP, as evidenced by the immediate myogenic differentiation of VLL progenitors upon transfection with Smad6. This led to the depletion of the local pool of muscle progenitors as they exited the epithelial layer. Concurrently, when the hypaxial myotome failed to enter the LPM, the RP remained within the epaxial domain and failed to extend upon reducing BMP activity in the LPM (Figure 8).

These findings are consistent with the concept of the DM/myotome complex driving the growth of the RP, as previously indicated by studies on mice lacking the DM gene Pax3 [2,69,70] or the myotomal muscle regulatory gene Myf5 [2,71,72]. In both cases, mispatterning or the absence of only the distal parts of the ribs was observed. Moreover, in chick embryos, it was shown that intercostal muscle extension into the somatopleura slightly precedes the growth of the rib primordium [1] until E6, when both ribs and myotomes reach the sternum. Along this line, LPM-derived BMP could also serve as an attractive signal for lateral sclerotomal and/or DM cells to enter the somatopleural environment.

Collectively, our results are consistent with the notion that graded Shh and BMP signaling pathways play crucial and distinct roles in orchestrating both early and later paraxial mesoderm development. They stand as pivotal factors in medio-lateral somite patterning, sclerotome induction, and subsequent morphogenesis of proximal and distal skeletal components, respectively (Figure 8). When considering the significant functions these morphogens also play in development of neural tube and muscle, it becomes apparent that only a handful of factors, such as Shh, BMP, and FGF8, are involved in coordinating the morphogenesis and subsequent functioning of the neuromuscular and skeletal systems.

## 4. Materials and Methods

### 4.1. Embryos

Fertilized quail (*Coturnix coturnix Japonica*) eggs were purchased from a commercial source (Moshav Mata), kept at 15 °C and then incubated at 38 °C until reaching the desired stages. Embryos were staged based on the number of somite pairs formed. All experiments were carried out at the flank level of the axis.

### 4.2. Expression Vectors and Electroporation

Expression vectors were: pCAGGS-GFP [73], XNoggin [74], pCAB-cSmad6 [75], and Hhip [76]. To achieve conditional expression, the latter was subcloned into pBI (pTRE) to yield PBI-TRE-Hhip and co-electroporated along with either pCAGGS-rtTA2s-M2 or pCAGGS-tTA plasmids [50,77]. To activate or inactivate expression of the above, respectively, doxycycline was added (50 μL of 1 ng/μL/egg/day). Significant GFP signal was detected post-electroporation as early as 6 h following antibiotic addition.

For electroporations, DNA (1–4 µg/µL) was microinjected into the center of flank-level epithelial somites (somites 20–25) of 25 somite-stage embryos. Electroporations were performed to the ventral half of epithelial somites (prospective sclerotome). To this end, the positive tungsten electrode was placed under the blastoderm in a location corresponding to the ventro-medial portion of epithelial somites on a length of about 5 segments, and the negative electrode was placed in a superficial, dorso-lateral position with respect to the same somites [18,78,79]. For VLL electroporations, plasmids were similarly injected into the somitocoel and current directed towards the dorsolateral aspect of the somites, as described [80] For LPM electroporations, DNA was microinjected into the coelomic cavity between both layers of the developing LPM. One electrode was placed above the ectoderm along the border between somites and LPM and the other electrode was placed underneath the endoderm at a slightly more lateral position. A square wave electroporator (ECM 830, BTX, Inc., Hawthorne, NY, USA, https://www.btxonline.com/products/electroporation-systems.html) was used. One pulse of 15 V for 6 msec was applied.

### 4.3. Immunohistochemistry

Embryos were fixed overnight at 4 °C with 4% formaldehyde in phosphate-buffered saline (PBS) (pH 7.4) followed by washings in PBS. For wholemount immunostaining, antibodies were diluted in PBS containing 1% Triton X-100 and 5% newborn calf serum and tissues were incubated overnight at 4 °C on a rotatory shaker. Next, they were washed twice in a large volume of PBS/1% Triton X-100 first for 10 min and then for 2 h at room temperature. Secondary antibodies were similarly diluted in PBS/1% Triton X-100/5% newborn calf serum and incubated overnight followed by repetitive washings. Embryo fragments were dehydrated in increasing ethanol solutions (30%,70%, 90%, and 100%, 10 min each) followed by toluene (2 times, 10 min each), then embedded in paraffin wax and sectioned at 8 μm. Paraffin was removed in Toluene and slides were rehydrated in decreasing ethanol solutions.

The following antibodies were used: rabbit anti-GFP (1:1000, Invitrogen, Thermo Fisher Scientific, Waltham, MA USA, https://www.fishersci.com/us/en/brands/IIAM0WMR/invitrogen.html, #A6455) or chicken anti-GFP (NOVUSBIO.com, Littleton, CO, USA, https://www.fishersci.com/us/en/catalog/search/products, NOVUSBIO, #NB100-1614, 1:500), SOX9 (Sigma, San Diego, CA, USA, https://www.sigmaaldrich.com/, #AB5535, 1:150) and mouse anti-desmin (1:200, Molecular Probes, Eugene, OR, USA, https://www.thermofisher.com/il/en/home/brands/molecular-probes.html, #10519). Monoclonal antibodies against Pax7 (PAX7-s, 1:20, DSHB, University of Iowa), phosphorylated Smad 1-5-8 (P-Smad, Cell Signaling, Danvers, MA, USA, https://www.cellsignal.com/, #CST-13820 mouse monoclonal, 1:500). Anti-Histone H3 (phospho S10, PHH3) was from Abcam, Cambridge, UK, https://www.abcam.com/, (mAbcam #14955, 1:500), and anti-Caspase 3 from Cell Signaling https://www.cellsignal.com/, (#9661, 1:200). Nuclei were visualized with Hoechst 33,258 (Sigma #14530, 125 ng/mL).

### 4.4. In Situ Hybridization

Embryos were fixed in Fornoy (60% ethanol, 30% formaldehyde, 10% acetic acid), dehydrated in ethanol/toluene, processed for paraffin wax embedding, and sectioned at 10 μm. Slides were rehydrated in toluene/ethanol/PBS, treated with proteinase K (1 µg/mL, Sigma Aldrich P2308) at 37 °C for 7 min, and then fixed in 4% formaldehyde at room temperature for 20 min. Next, slides were washed in PBS followed by 2× SSC and hybridized in hybridization buffer (1× salt solution composed of 2 M NaCl, 0.12 M Tris, 0.04 M NaH_2_PO_4_·2H_2_O, 0.05 M Na_2_HPO_4_, 0.05 M EDTA, pH 7.5], 50% formamide, 10% dextran sulfate, 1 mg/mL Yeast RNA, 1× Denhardt solution) containing 1 μg/mL DIG labeled RNA probes (prepared with a DIG RNA labeling mix, Roche, Basel, Switzerland, 11277073910) for overnight at 65 °C in a humid chamber. Post-hybridization, slides were rinsed in a rotating incubator with 50% formamide, 1× SSC, 0.1% Tween 20 until coverslips dropped and then an additional wash for 1 h was performed followed by 2 washes in MABT (10% Maleic acid 1 M pH 7.5, 3% NaCl 5 M, 0.1% Tween 20) and preincubation in MABT/2.5% FCS. Anti-DIG-AP antibody (1/1000, Roche 11093274910) diluted in MABT+2% BBR+20% FCS was then added for overnight at room temperature. This was followed by rinsing in MABT and then in NTMT (2% NaCl 5 M, 10% Tris HCl 1 M pH9.5, 5% MgCl_2_ 1 M, 0.1% Tween20), and then incubation in NTMT + 1:200 NBT/BCIP Stock Solution (Sigma-Aldrich, St. Louis, MO, USA, 11681451001) at 37 °C until the AP reaction was completed. The following probes were employed: *SOX9* (from M. Cheung), *PAX1*, *PAX9*, *NKX3.2*, *MEOX2* (from R. Reshef).

### 4.5. Data Analysis and Statistics

Several variables were measured in both control and treated sides using Adobe Photoshop CS6 (version 13) and Image J software (https://imagej.net/ij/) on photographed sections. Values are expressed as the ratio between treated to contralateral intact side ± SEM.

The following parameters were monitored: VB size was measured as the area delineated by notochord, ventral neural tube, and myotome, as depicted in Figure 3A. VB thickness in the dorso-ventral extent was determined by drawing a straight line connecting the ventro-lateral corner of the neural tube and the upper coelomic border (Figure 3C); intensity of *SOX9* expression in VB was quantified within a defined area drawn on both sides of the paranotochordal region (Figure 3E). The area of the RP encompassed the *SOX9*+ tissue that extended beyond the coelomic border into the LPM, and the extent of ingrowth was monitored as a straight line connecting the upper coelomic border with the distal edge of the RP (Figure 3F). The SP area, defined by *SOX9* ventral to the RP, was similarly measured (Figure 6B). *SOX9* intensity in the RP or SP was quantified within the entire area delimited for size measurements.

To quantify the lateral deviation of the notochord from the midline, a straight line connecting the midline of the ventral tube lumen and floor plate all the way through the notochord, was drawn perpendicular to the ventral domain of bilateral *SOX9* expression in the ventricular layer (Figure 4A,B). The area of the treated and control hemi-notochords was then measured and expressed as [T − C]/[T + C].

The number of sections per embryo analyzed for each of the above parameters ranged between 10 and 25. The number of PHH3 or Caspase 3-positive nuclei was monitored in 10–25 sections per embryo. Further details of quantifications are summarized in Supplementary Table S1.

Images were photographed using a DP73 (Olympus, Tokyo, Japan) cooled CCD digital camera mounted on a BX51 microscope (Olympus) with Uplan FL-N 20×/0.5 and 40×/0.75 dry objectives (Olympus). For quantification, images of control and treated sections were photographed under the same conditions. For figure preparation, images were exported into Photoshop CS6 (Adobe). If necessary, the levels of brightness and contrast were adjusted to the entire image and images were cropped without color correction adjustments or γ adjustments. Final figures were prepared using Photoshop CS6.

Significance of results was determined using the non-parametric Kruskal–Wallis test with post-hoc Mann–Whitney. For Figure 1 and Supplementary Figure S1 where 2 study groups were compared, the non-parametric Mann–Whitney test was implemented.

In general, tests applied were two-tailed, and a *p*-value of 0.05 or less was considered statistically significant. However, in the experiments shown in Figure 3 and Figure 4, the significance level was corrected according to Bonferroni for multiple paired comparisons. Data were analyzed using GraphPad Prism version 9. The number of embryos analyzed for each treatment (*n*) and *p*-values can be found in the corresponding Legends.

## Data Availability

Data is contained within the article and Supplementary Materials.

## Supplementary Materials

The following supporting information can be downloaded at: https://www.mdpi.com/article/10.3390/ijms25115602/s1.
