# Supplementary material for: A Spatio-Temporal-Dependent Requirement of Sonic Hedgehog in the Early Development of Sclerotome-Derived Vertebrae and Ribs
Source: Int J Mol Sci. 2024 May 21;25(11):5602. doi: 10.3390/ijms25115602 (PMC11171667; doi:10.3390/ijms25115602)
Supplement: Supplementary file 1 [file ijms-25-05602-s001.zip › ijms-2989064-supplemenataryfigures.pdf]

Legends to Supplementary Figures and Figures

Supplementary Figure S1

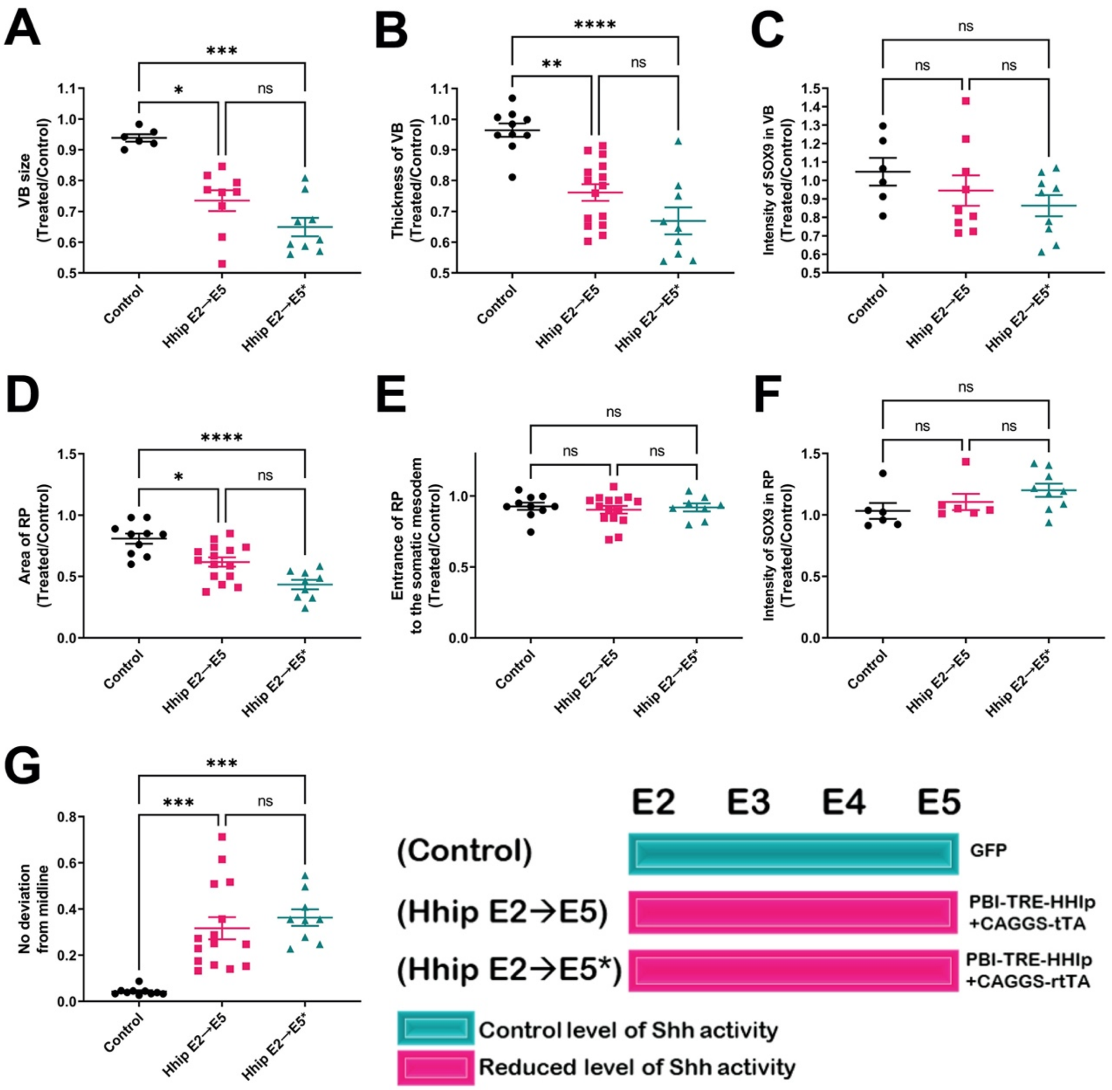

Supplementary Figure S1- Inhibition of Shh by two conditional plasmids yield similar effects on skeletal development

To inhibit Shh from E2 until E5, 2 kinds of plasmids were implemented. PBI-TRE-Hhip+CAGGS-tTA, a version of Hhip that is active in absence of added doxycycline (HhipE2-E5), or PBI-TRE-Hhip+CAGGS-rtTA, a version of Hhip that is inactive unless doxycycline is added, followed by immediate treatment with the antibiotic (Hhip E2-E5*). (A-G) Quantifications of the effects of both plasmids compared with control GFP on selected parameters of skeletal development. For control vs. Hhip E2-5, or Hhip E2-5* , respectively, the number of embryos analyzed was: 6,9,9 embryos for VB size and SOX9 intensity in VB and RP; 10, 15, 9 for VB thickness, RP area and entrance into LPM and for notochord deviation. *P<0.05, **P<0.01, ***P<0.001, ****P<0.0001, ns-not significant.

Supplementary Figure S2

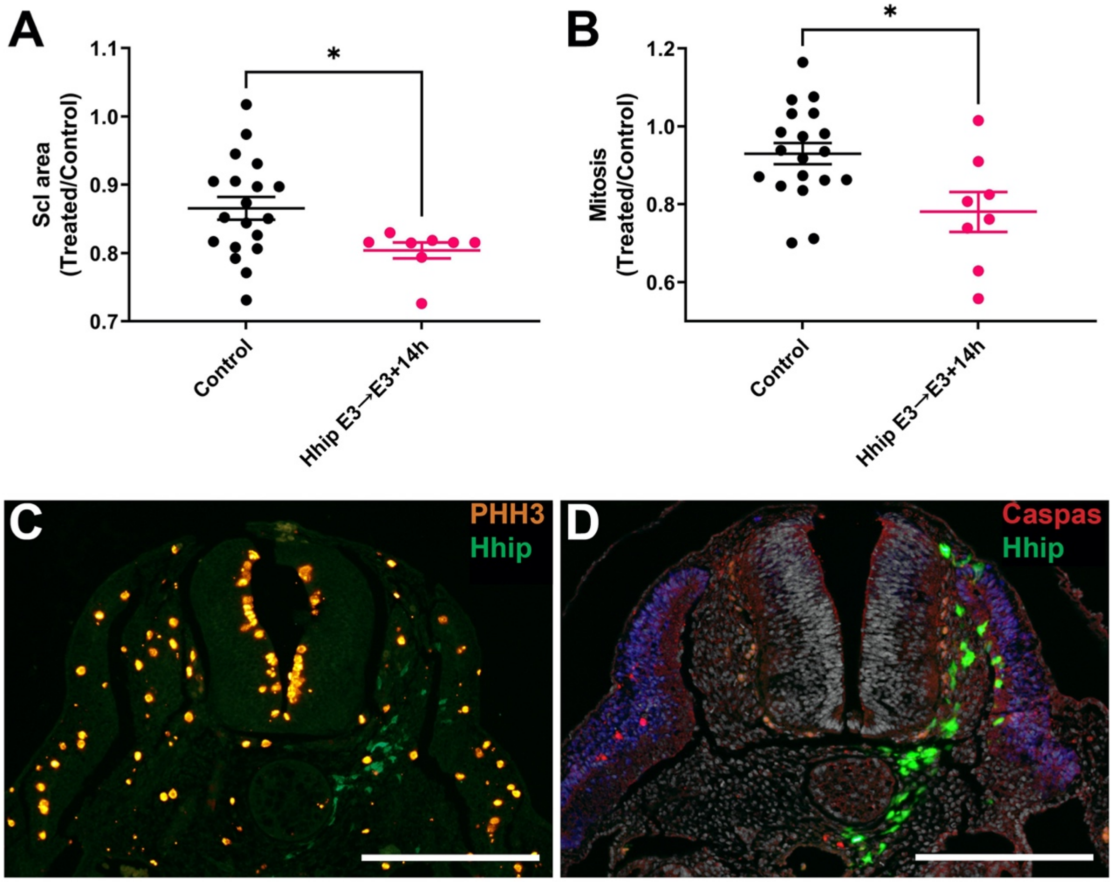

<u>Supplementary Figure S2- Reduced sclerotome area and cell proliferation, but not survival, upon activation of Hhip at E3</u>

Control GFP or Hhip were electroporated at E2 and activated at E3 for 14 hours. (A) Quantification of sclerotome area. (B) Quantification of PHH3+ mitoses. (C,D) Transverse sections showing reduced number of mitoses in the treated (right) compared to contralateral side (C), and the virtual absence of caspase+ cells in either treated or control sides at this stage (D). N=19 and 8 embryos measured for control or Hhip, respectively. Bar= 200μM.
